# 313. Impact of a Project Firstline Educational Campaign on Hospital Acquired Infection Rates Pre/Post Intervention

**DOI:** 10.1093/ofid/ofaf695.109

**Published:** 2026-01-11

**Authors:** Shelley Kon, Christine M Gunter, Heidi Hough, Randi Craig, Linda M Gonzalez, Allison E Boyrer, Mary T Bessesen

**Affiliations:** Rocky Mountain Regional Veterans Affairs Medical Center, Aurora, Colorado; Rocky Mountain Regional Veterans Affairs Medical Center, Aurora, Colorado; VA Eastern Colorado Health Care System, 80045, Colorado; Rocky Mountain Regional Veterans Affairs Medical Center, Aurora, Colorado; VA Eastern Colorado Health Care System, 80045, Colorado; VA Eastern Colorado Health Care System, 80045, Colorado; Rocky Mountain Regional Veterans Affairs Medical Center, Aurora, Colorado

## Abstract

**Background:**

Project Firstline (PFL) is a national training collaborative for healthcare infection prevention and control (IPC). PFL was created by the Centers for Disease Control and Prevention, in partnership with the American Academy of Pediatrics and the American Nurses Association. PFL provides innovative content about fundamental IPC principles.

Average Hospital Acquired Infection Rate per Year

**Figure 1:**
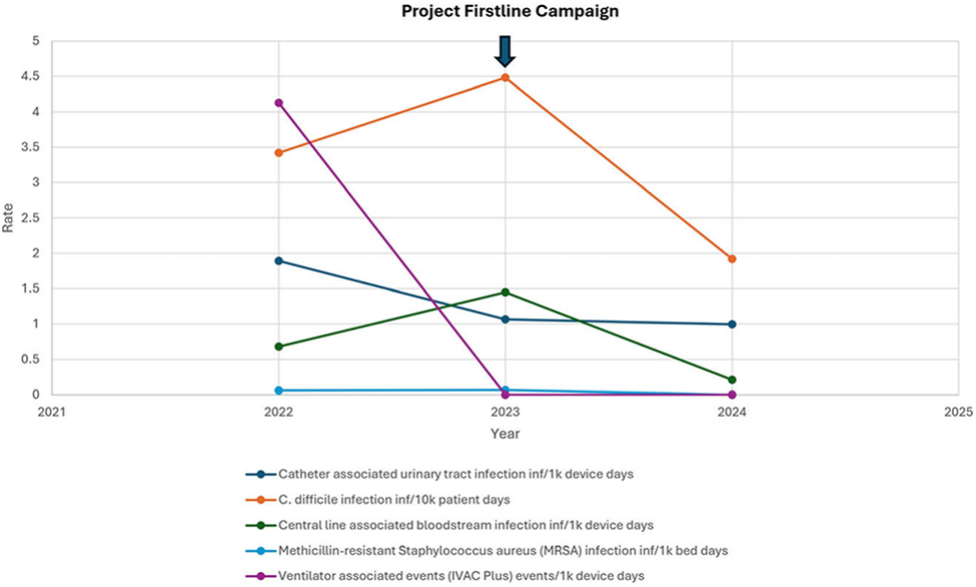
Average 12-month Hospital Acquired Infection (HAI) rate by calendar year in the year prior (baseline), intervention year, and year post intervention.

**Methods:**

The IPC team partnered with the Post-Baccalaureate Registered Nurse Residency Program (PB-RNR) at a 180 bed, academically affiliated Veterans Affairs (VA) hospital to implement a multifaceted educational campaign utilizing PFL resources from January 2023- December 2023. Three PB-RNR nurse residents trained as IPC champions. PFL assets were incorporated into unit huddle boards, nursing orientation, a skills fair and micro education sessions on medical/surgical, spinal cord injury, intensive care units and a 40-bed community living center. PFL resources were also added to an internal website, incorporated into the facility wide communications email and posted onto the VA’s employee Facebook site. Average 12-month Healthcare-Associated Infection (HAI) rates were compared at baseline (2022) and post intervention (2024). Of note, other quality improvement projects took place during the PFL campaign.

**Results:**

536 staff attended the educational sessions, including micro education sessions, nursing orientation and skills fair taught by IPC staff and the nurse resident champions. The audience primarily consisted of nurses, followed by nursing assistants and physicians. The facility-wide email had 10,260 unique views. Four targeted unit-based huddle boards were created. The IPC internal website had 1,219 views. The facility wide average 12-month Catheter Associated Urinary Tract Infection (CAUTI) rate went from 1.894 to 0.996 per 1,000 device days, a 47% decrease. The facility wide rolling 12-month Central Line Associated Bloodstream Infection (CLABSI) rate went from 0.681 to 0.209 per 1,000-line days, a 69% decrease. The facility wide rolling 12-month hospital onset *Clostridioides difficile* infection rate went from 3.422 to 1.92 per 10,000 patient days, an 44% decrease. (See Figure 1).

**Conclusion:**

Implementation of an educational campaign utilizing PFL resources was associated with a decrease in facility wide HAI rates.

**Disclosures:**

All Authors: No reported disclosures

